# Frequency-Aware Summarization of Resting-State fMRI Data

**DOI:** 10.3389/fnsys.2020.00016

**Published:** 2020-04-07

**Authors:** Maziar Yaesoubi, Rogers F. Silva, Armin Iraji, Vince D. Calhoun

**Affiliations:** ^1^The Mind Research Network, Albuquerque, NM, United States; ^2^Tri-institutional Center for Translational Research in Neuroimaging and Data Science (TReNDS), Georgia State University, Georgia Institute of Technology, Emory University, Atlanta, GA, United States; ^3^Electrical and Computer Engineering Department, The University of New Mexico, Albuquerque, NM, United States

**Keywords:** resting-state fMRI, time-frequency analysis, dimension reduction, canonical correlation analysis, independent component analysis, functional connectivity, Hilbert transform

## Abstract

Many brain imaging modalities reveal interpretable patterns after the data dimensionality is reduced and summarized via data-driven approaches. In functional magnetic resonance imaging (fMRI) studies, such summarization is often achieved through independent component analysis (ICA). ICA transforms the original data into a relatively small number of interpretable bases in voxel space (referred to as ICA spatial components, or spatial maps) and corresponding bases in the time domain (referred to as time-courses of corresponding spatial maps) In this work, we use the word “basis” to broadly refer to either of the two factors resulting from the transformation. A precise summarization for fMRI requires accurately detecting co-activation of voxels by measuring temporal dependence. Accurate measurement of dependence requires a proper understanding of the underlying temporal characteristics of the data. One way to understand such characteristics is to study the frequency spectrum of fMRI data. Researchers have argued that information regarding the underlying neuronal activity might be spread over a range of frequencies as a result of the heterogeneous temporal nature of the neuronal activity, which is reflected in its frequency spectrum. Many studies have accounted for heterogeneous characteristics of the frequency of the signal by either directly inspecting the contents of frequency domain-transformed data or augmenting their analyses with such information. For example, studies on fMRI data have investigated brain functional connectivity by leveraging frequency-adjusted measures of dependence (e.g., when a correlation is measured as a function of frequency, as with “coherence”). Although these studies measure dependence as a function of frequency, the formulation does not capture all characteristics of the frequency-based dependence. Incorporating frequency information into a summarization approach would enable the retention of important frequency-related information that exists in the original space but might be lost after performing a frequency-independent summarization. We propose a novel data-driven approach built upon ICA, which is based on measuring dependence as a generalized function of frequency. Applying this approach to fMRI data provides evidence of existing cross-frequency functional connectivity between different areas of the brain.

## Introduction

Neuronal activity is highly dynamic and covers a range of temporal scales (Jensen and Colgin, 2007; Baria et al., 2011). The dynamic activity results from the anatomy of the brain itself, as well as the nature of external and internal stimuli in the brain (Von Stein et al., 2000). Brain imaging can be thought of as a modulated observation of neuronal activities. That is, different modalities are subject to different modulation characteristics due to the physical and physiological principles of the brain and the imaging modality. To be able to link the observed signals with the underlying neuronal activities accurately, we need complete knowledge of the modulation characteristics.

However, comprehensive knowledge of such characteristics is seldom available. For example, in fMRI, modulation is characterized by a hemodynamic response function (HRF; Logothetis et al., 2001). The modulated signal is modeled as the convolution of the HRF with the underlying neuronal activities, referred to as blood-oxygenation-level dependent (BOLD) signal. Because the HRF is a slow-varying function relative to the underlying neuronal oscillation, information regarding the neuronal activity is assumed to be in the lower frequency part of the BOLD signal spectrum.

**FIGURE 1:**
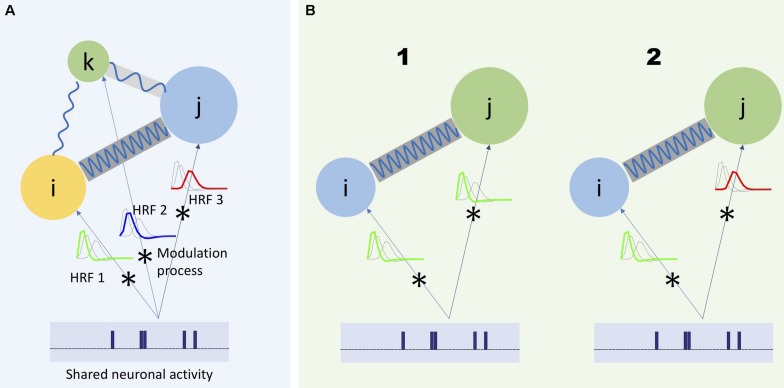
Three regions of the brain (represented by colored circles) has a unique modulation process modeled as the convolution of the shared neuronal activity with its corresponding HRF. As it is shown in panel **(A)**, each HRF has a unique frequency spectrum, which would lead each region to capture different frequency spectrum portions of the same neuronal activity. Moreover, in a more general scheme, the communication between different regions of the brain can also occur at different frequency bands (shown by blue waves with different frequencies connecting the circles) (Baria et al., 2011). Any study to investigate the actual characteristics of the underlying neuronal activity should consider such heterogeneous aspects of the captured activities with respect to the frequency. For example, when it is required to measure dependence between two regions, it would be beneficial to measure such dependence as a function of frequency. **(B1)** Two regions share the same neuronal activity and the same HRF (represented as a low-pass filter). Hence, dependence should be measured as a function of one shared frequency between the regions (in-between frequency) and **(B2)** as a function of different frequencies between the regions (cross-frequency dependence).

However, the assumptions of the HRF function conflict with available evidence (Chang and Glover, 2010; Gohel and Biswal, 2015; Yaesoubi et al., 2015a). For example, considering HRF as a slow-varying function does not explain all the spectral variation of fMRI data (Gohel and Biswal, 2015). Additionally, research has called into question traditional models of HRF as a time-invariant modulation filter. There have been observations of neuronal-related activity in the higher frequency part of the spectrum, including evidence that such activity varies in time (Chang and Glover, 2010; Yaesoubi et al., 2015a). For example, in the latter study, given the repetition time (TR) of scanner being 2 s, evidence of dynamic functional connectivity occurring at frequencies higher than 0.15 Hz has been provided. Because neuronal activity can have different rates of change, we expect that different parts of the BOLD signal spectrum will carry different information regarding the underlying neuronal activity. Figure 1A illustrates these considerations.

**FIGURE 2:**
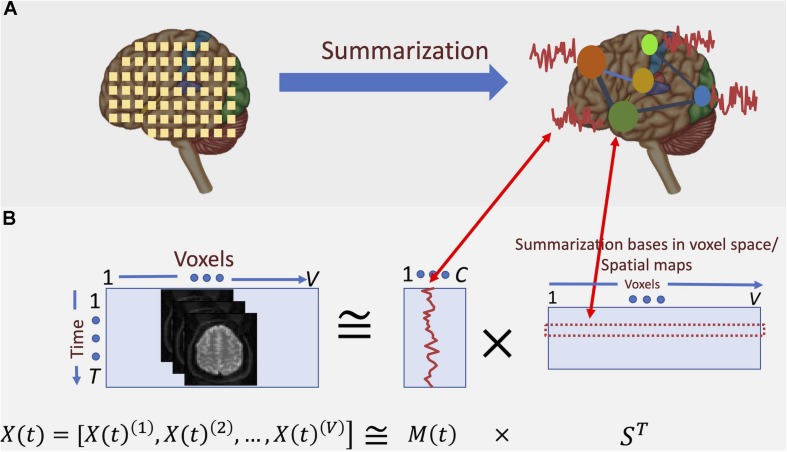
Conceptual depiction of summarization procedure. **(A)** fMRI captures the data at voxel-level. However, groups of voxels correspond to specific functional regions of the brain and, hence, have similar activation patterns. Summarization approaches such as ICA aim at finding these groups and the corresponding activation patterns. **(B)** Here, the summarization is formulated as a blind low-rank matrix decomposition (“blind” means that neither of the underlying multiplicative factors is known). In the case of spatial ICA, the major constraint of such decomposition is that the “bases” in voxel space (i.e., the spatial maps) are maximally statistically independent from each other. It is shown that the spatial independence assumption yields spatial maps that parcel the brain into functionally related networks (or groups) of voxels.

Consequently, an accurate summarization of fMRI data needs to consider the frequency spectrum of the BOLD signal. For example, a standard analysis for studying functional segregation (Tononi et al., 1994) of the brain is determining collections of voxels with similar activity patterns over time, which results in parcellation of the brain into functional regions or units (Wang et al., 2015; Gordon et al., 2017; Iraji et al., 2019b). When these regions are determined via a data-driven approach, voxel-level data is summarized into a lower-dimensional subspace. Figure 2 illustrates this concept: we can represent the summarization as the transformation of bases of the data from its original space, followed by dimension reduction. However, such summarization is independent of the underlying frequency information of the data.

In this work, we propose a novel method to incorporate the frequency spectrum of the data into independence component analysis (ICA) as a commonly used summarization approach for studying fMRI data.

Independence component analysis consists of a bases transformation and dimension reduction via principal component analysis (PCA), followed by the next bases transformation via ICA (Calhoun et al., 2001). The transformed spatial bases correspond to collections of voxels with similar activity patterns, widely referred to as spatial maps. These spatial bases parcel the brain into spatially independent (segregated) components, and each component includes a network of spatially distributed voxels with similar activity patterns (referred to as a functional network; Calhoun and Adali, 2012).

Independence component analysis is a blind source separation method formulated as a low-rank matrix decomposition of the input data matrix X_T×V_ into two matrices, M(t)_T×C_ and S_V×C_ as follows:

(1) $$\text{X}\left(\text{t}\right)≅\text{M}\left(\text{t}\right)\times {\text{S}}^{⊤}$$

where T is the time dimension, V is the voxel dimension, and C is the reduced dimension (a.k.a., the subspace dimension or number of components). C is also the rank of the decomposition, and typically C ≪ T and V. This decomposition is solved based on maximizing the independence across the columns of S (the spatial maps). The spatial maps are referred to as the underlying independent sources of the observed data. M(*t*) is referred to as the mixing matrix, which represents the temporal profiles of the spatial maps, referred to as the time-courses (TCs). C is also the rank of the decomposition, and typicallyC ≪ T and V.

The input fMRI data are voxel-wise time-series, expressed as X(t) = [X(t)^1^, X(t)^2^, …, X(t)^V^]. ICA is used to find maximally independent spatial maps (hence referred to as spatial ICA or sICA)^1^ and a mixing matrix M(*t*) representing TCs. This decomposition can estimate temporal dependence between functionally segregated functional regions (i.e., functional connectivity), as reflected in M(*t*). Figure 2B depicts how such decomposition translates into functional parcellation of the brain.

Based on the fact that the BOLD signal has heterogeneous frequency characteristics, any particular summarization could be modified to account for its frequency content. Since an essential element in these analyses is to measure the dependence of activity among voxels or collections of voxels, any modification should also measure dependence as a function of frequency. Various studies are addressing this goal by leveraging Fourier or time-frequency transformation (Cordes et al., 2001; Chang and Glover, 2010). In these studies, the input signal is transformed into Fourier or wavelet domains, and further analyses, including dependence estimation, are performed therein. For example, coherence has been used to study activity dependence between different regions of the brain as a function of frequency (Cordes et al., 2001), and it has enabled the detection of varying dependence characteristics across various regions of the brain. However, coherence lacks the notion of time and, consequently, it is unable to characterize the dynamics of dependence. More recent studies have leveraged time-frequency transformation to measure dependence as a function of both time and frequency (Chang and Glover, 2010; Yaesoubi et al., 2015a; Faghiri et al., 2019). These studies enable studying the time-varying characteristics of functional network connectivity (referred to as “dynamic connectivity”) along with frequency characteristics. For example, Yaesoubi et al., used wavelet transform coherence to measure dynamic connectivity between ICA-driven functional networks as a function of frequency (Yaesoubi et al., 2015a). Such an approach is useful when two different networks of the brain share the same underlying neuronal activity, and both modulate the activity with the same narrow-band modulation kernel (Figure 1B1 illustrates this). In such a scenario, traditional approaches that do not consider frequency information (e.g., correlation-based analyses) would blur the actual dependence with unrelated variations in other parts of the frequency spectrum of the networks’ TCs. In another reasonable scenario, the two networks might have different modulation process on the shared neuronal activity (Figure 1B2). In such a situation, when dependence is measured as a function of the same shared frequency for both TCs, the actual dependence might not be estimated correctly. Consequently, to capture the true nature of the dependence of underlying neuronal activity, dependence should be measured as a generalized function of two frequencies. Dependence as a function of one frequency is usually referred to as in-between frequency dependence (Figure 1B1), and when dependence is a function of two unequal frequencies, it is commonly referred to as cross-frequency dependence (Figure 1B2); the latter is largely missing in fMRI studies.

In this study, we propose a new approach to summarize resting-state fMRI data based on measuring the in-between and cross-frequency dependence and use both types of dependence estimations to estimate spatial maps as summarization bases in voxel space. For the time-frequency transformation of the input resting-state fMRI (rs-fMRI) data, we use the Hilbert transform. The Hilbert transformation estimates the instantaneous frequency content of the signal. Next, we use multiset canonical correlation analysis (MCCA; Li et al., 2009) to summarize voxel-level fMRI data (via basis transformation and data reduction) into several spatial maps and corresponding TCs. Because MCCA is applied in the time-frequency transform domain, our summarization approach accounts for existing in-between and cross-frequency estimations of the dependence of the spatial maps.

## Materials and Methods

We estimate spatial maps by modifying the ICA-based summarization approach to incorporate frequency information of BOLD signal. We adjust the ICA-based summarization approach by measuring dependence as a general function of frequency, accounting for both in-between and cross-frequency dependences.

Naturally, the first step is to augment the voxel-level time-series with frequency information. This augmentation can be achieved by time-frequency transformation of the input time-series. There are a few choices for this step, including whether to use the wavelet transform, the short-time Fourier transforms (STFT), and the Hilbert transform (Bruns, 2005). Among these three, the wavelet transform is a multi-resolution-based transformation, which means that for different frequencies (or scales in wavelet’s terminology), we have different time-resolutions. However, that would make the estimation of cross-frequency dependence less straight-forward. STFT is basically the Fourier transform of a windowed signal, which means that the bases in the frequency domain are pre-defined by the Fourier bases. The Hilbert transform, on the other hand, by-passes the windowing operation and instead requires narrow band-pass filtering of the signal before Hilbert domain mapping. Each band-pass filtered version of the signal (over different frequency bands) leads to a different Hilbert transformation at the same temporal resolution of the original signal. Combined, the Hilbert transformations constitute a convenient time-frequency representation of the input signal.

Because Hilbert transformations have the same temporal resolution at different frequency bands and do not require windowing, in contrast to STFT, we adopt it as the time-frequency transformation of choice for this work.

Initially, the input time-series of voxel v(X(T)^(v)^) is band-pass filtered in selected “narrow” frequency bands, and each band-passed version is transformed to the analytic form A(t)e^∅(t)^, where A(t) is the instantaneous amplitude of the filtered signal and ∅(t) is its instantaneous phase.

Mathematically speaking, let X_*nbp*_(t)^(v)^ represent a narrow band-passed version of original input time-series X(t)^(v)^ belonging to voxel v. Hilbert transform of this time-series is defined as:

(2) $$\begin{array}{c} H\left[{\text{X}}_{nbp}{\left(\text{t}\right)}^{\left(\text{v}\right)}\right]=p.v.\int_{-\infty }^{+\infty }\frac{{\text{X}}_{nbp}{\left(\text{t}-\tau \right)}^{\left(\text{v}\right)}}{\pi \tau }\partial \tau \end{array}$$

where *p*.*v*. is the Cauchy principal value of above convolution. For more information you can refer to Hildebrand (1949). For our application, we used MATLAB implementation of Hilbert transform for discrete time-series, which used the algorithm proposed in Marple (1999) to estimate the above integration for a discrete time-series.

The analytic form of the band-passed time-series X_*nbp*_(t)^(v)^ is now defined as following:

(3) $${\text{X}}_{nbp}{\left(t\right)}^{\left(\text{v}\right)}+\text{j}H\left[{\text{X}}_{nbp}{\left(t\right)}^{\left(\text{v}\right)}\right]=A\left(\text{t}\right){\text{e}}^{\emptyset \left(\text{t}\right)}$$

**FIGURE 3:**
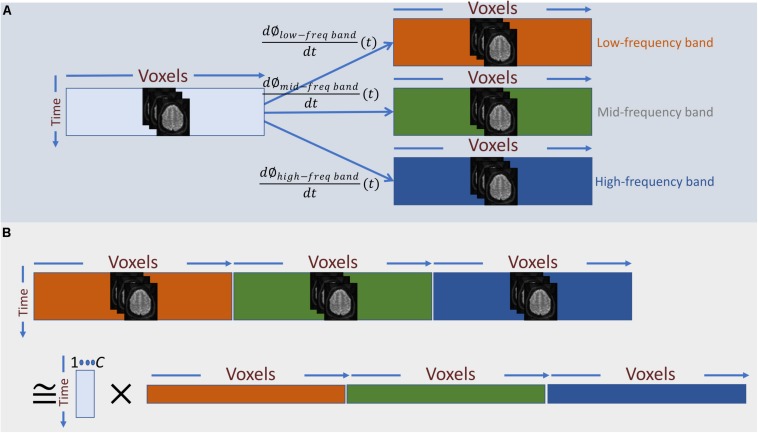
**(A)** Frequency information is added to the temporal activation pattern of each voxel via Hilbert-based band-passed time-frequency transformation and instantaneous frequency estimation. **(B)** In a naïve approach, we can treat the instantaneous frequency representation of each band as a new set of independent voxel measurements and perform summarization on the spatially concatenated data. The main draw-back of this approach is that it barely captures any dependence between instantaneous frequencies across different bands.

The derivative of the instantaneous phase (i.e., $\frac{\text{d}\emptyset }{\text{dt}}\left(\text{t}\right)$) is the instantaneous frequency, which we use as temporal representation of the original time-series at the given frequency-band (Figure 3A). For band-pass filtering, we used a seventh order Butterworth digital filter at six different narrow frequency ranges, all in Hz: 0.0025–0.0375, 0.0375–0.0750, 0.0750–0.1125, 0.1125–0.1500, 0.1500–0.1875, and 0.1875–0.2250.

Having a time-frequency representation of the time-series of each voxel as the input data, the next step is to summarize the data using spatial ICA. In general, the summarization step can be considered as a low-rank matrix decomposition (as in Eq. 1), A naïve way to apply this decomposition into the augmented data is to treat each band-specific time-series as a new set of voxel time-series and perform the summarization on the concatenated data across voxel-space (Figure 3B). However, the primary assumption of this approach is that cross-frequency and in-between frequency dependence measurements are directly comparable. However, t in-between and cross-frequency might explain very different amounts of variance in the data. For example, the power of the underlying neuronal activity may vary as a function of frequency, and the HRF can change over different regions of the brain, which imply that cross-frequency and in-between frequency dependence measures might not be in a comparable scale. Hence, the significance level of dependence would vary across frequency bands, rendering this approach inadequate. Unless an accurate adjustment is made to the measure of dependence such that all the dependence measurements are directly comparable, this naïve approach would be equivalent to running ICA separately on each band-specific data.

**FIGURE 4:**
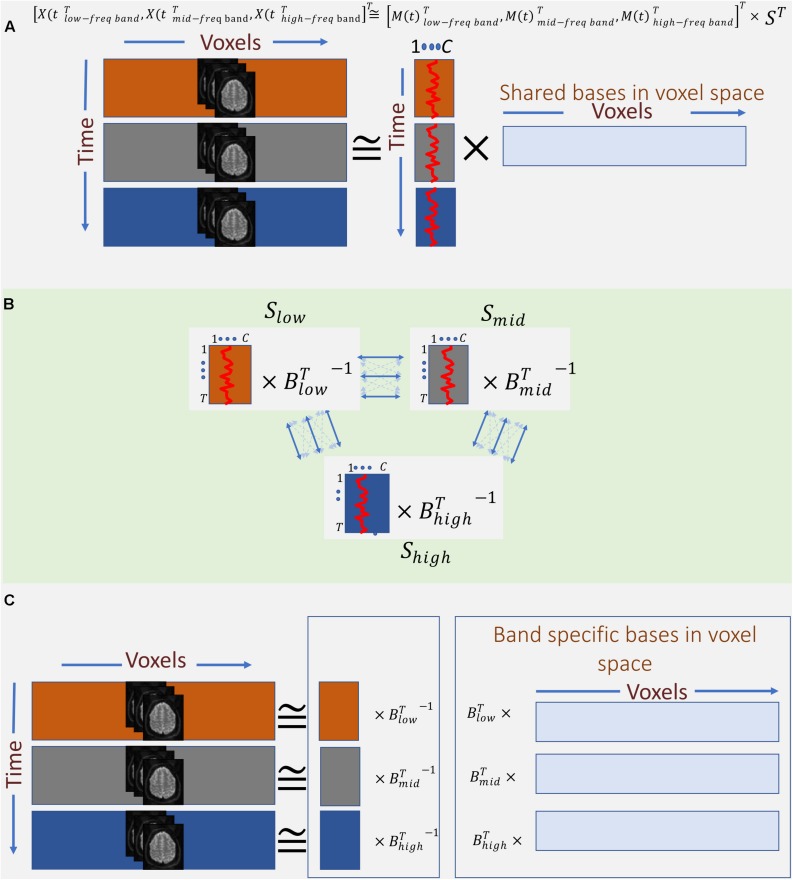
Outline of the proposed summarization approach incorporating in-between and cross-frequency dependence among voxel activation patterns. **(A)** First, band-specific data are concatenated along with the time domain, and the first summarization step is applied to find shared spatial bases formed based on their frequency properties. **(B)** Second, band-specific bases in the time domain are transformed such that corresponding bases across bands are maximally correlated. **(C)** Finally, the two transformations **(A, B)** are blended to find bases along with voxel domain (spatial maps), which are derived from both in-between and cross-frequency dependence.

Therefore, instead of concatenating band-specific data across voxel-space, we consider the concatenation of the data along the temporal domain (Eq. 4 and Figure 4A). This allows us to find a common space (shared bases in voxel space) across different frequency bands. However, this approach only leverages the in-between frequency (not cross-frequency) information to estimate the shared bases. In other words, the estimated spatial map reflects the degree to which voxels in that map show some in-between dependence. Essentially, this step outputs summarization bases in voxel space (i.e., spatial maps) that are shared across bands, whereas the corresponding temporal signatures (TCs) carry band-specific information. *Post hoc* analysis can be used to inspect the temporal signature of each frequency band from the aggregate time-course corresponding to a given spatial map. For example, estimating the amount of power in each band allows us to indicate whether dependence occurring at any of the bands dominates over the others, or if the dependence is more evenly distributed over bands. When the power of a given spatial map’s time-course is concentrated within a specific band, it indicates that the spatial map and its associated regions correspond to that specific band and share the same in-between frequency. Likewise, when the power of the time-course is spread over multiple bands, the spatial map highlights areas which in-between dependence co-occurs over multiple bands. Moreover, cross-correlating these band-specific TCs can also yield extra information about cross-frequency dependence. However, because all bands share the same spatial map, this assessment is restricted to the areas contained in that map. This considerably limits the approach and prevent assessments outside the shared spatial map. Nonetheless, the ability to identify bases according to aggregate in-between frequency dependence justifies further experimentation of this approach as follows:

Assuming, for ease of explanation, that we only use three frequency bands for the time-frequency Hilbert transformation, namely, low, mid, and high^2^ bands. The transformed data is a temporal concatenation of the original data as follows:

(4) $$X\left(t\right)={\left[X{\left(t\right)}_{\mathrm{low}\mathrm{band}}^{⊤},X{\left(t\right)}_{\mathrm{mid}\mathrm{band}}^{⊤},X{\left(t\right)}_{\mathrm{high}\mathrm{band}}^{⊤}\right]}^{⊤}$$

This temporally concatenated data is then approximated by ICA with a low-rank decomposition as follows:

(5) $$X(t)≅\begin{array}{l} {\left[M{\left(t\right)}_{\;}{}_{\mathrm{low}\;\mathrm{band}}^{⊤},M{\left(t\right)}_{\;}{}_{\mathrm{mid}\;\mathrm{band}}^{⊤},M{\left(t\right)}_{\;}{}_{\mathrm{high}\;\mathrm{band}}^{⊤}\right]}^{⊤}\times S^{⊤} \end{array}$$

Just as in Eq. 1, M(t)_i_ represent the mixing matrices (or summarization bases in temporal domain) and S represents a matrix containing C (C ≪ T, V) independent spatial sources.

Thus far, we have captured spatial maps as bases in voxel space based on dependence as function of one frequency (i.e., in-between dependence). An important contribution of this work is also incorporating cross-frequency dependence into the formation of the spatial maps. This is achieved by leveraging MCCA. The goal of MCCA is to find underlying correlated sources across multiple data-sets. Thus, one dimension must match across data-sets in order to measure correlations (linear dependence) among them. Satisfying this requirement, MCCA finds a separate transformation for each data-set such that corresponding bases along the matching dimension have maximum correlation across data-sets and, at the same time, are uncorrelated with other bases both within and across data-sets. In this study, we treat the mixing matrices M(t)_*i*_ as the multi data-sets in MCCA, which solves for blind decomposition of each M(t)_*i*_ as follows (Figure 4B):

(6) M(t)i⊤=Bi×S(t)i⊤′

i∈{lowband,midband,highband}

Similar to Eq. 1, $\text{S}{\left(\text{t}\right)}_{\text{i}}^{{}^{\prime }}{}^{⊤}$ are the sources (but here along temporal domain) with properties enforced by MCCA as follows:

$\forall \text{i}𝔼\left[\text{S}{\left(\text{t}\right)}_{\text{i}}^{{}^{\prime }}⊤\text{S}{\left(\text{t}\right)}_{\text{i}}^{{}^{\prime }}\right]=\text{I}$, where I is the identity matrix, meaning the temporal sources are uncorrelated within each data-set, and $\forall \text{i}\neq \text{j}𝔼\left[\text{S}{\left(\text{t}\right)}_{\text{j}}^{{}^{\prime }}⊤,\text{S}{\left(\text{t}\right)}_{\text{i}}^{{}^{\prime }}\right]=\Lambda$, where Λ is a diagonal matrix and the diagonal elements represent the degree of dependence across corresponding sources for each pair of data-sets. Thus, temporal sources are only correlated with their corresponding temporal sources from other bands. This temporal dependence effectively captures the desired cross-frequency dependence, which then drives the formation of the temporal bases.

Finally, we combine the two summarization steps to estimate spatial maps which are formed based on both in-between and cross-frequency dependence as follows:

$$\text{X}{\left(\text{t}\right)}_{\text{i}}=\text{M}{\left(\text{t}\right)}_{\text{i}}\times {\text{S}}^{⊤}=S{\left(\text{t}\right)}_{\text{i}}^{{}^{\prime }}\times {\text{B}}_{\text{i}}^{⊤}\times {\text{S}}^{⊤}$$

In the results section below, we report ${\text{B}}_{\text{i}}^{⊤}\times {\text{S}}^{⊤}$ as band-specific spatial maps (Figure 4C).

### Data

We analyzed resting-state scans of 200 all healthy participants ages 12–35 years (mean = 21). Participants reported their gender as either “men” (*n* = 90) or “women” (*n* = 110). Subjects were given written informed consent, following institutional guidelines approved by the Institutional Review Board of the University of New Mexico. Scans had a minimum duration of 5 min and 4 s with a TR of 2 s, resulting in 152 volumes. Excess volumes of subjects with longer duration were discarded. Also, the first four volumes were discarded to avoid T1 equilibration effect. All participants have maximum translation less than 1.5 mm and with spatial correlation to EPI template greater than 0.93. All subjects were instructed to keep their eyes open.

### fMRI Acquisition and Pre-processing

Same 3-T Siemens Trio scanner with a 12-channel radiofrequency coil, was used for all the subjects. Gradient echo-planar imaging (EPI) sequence with echo time (TE) of 29 ms and TR of 2 s were used. Other scanning parameters were flip angle = 75° with a slice thickness of 3.5 mm and a slice gap of 1.05 mm. The field of view was 240 mm, and voxel size was 3.75 mm^3^ × 3.75 mm^3^ × 4.55 mm^3^. A standard SPM pre-processing pipeline^3^ was used for pre-processing of the functional images. Steps included realignment, motion correction using the INRIAlign, slice-timing correction, spatial normalization to Montreal Neurological Institute space and resampling to 3 mm^3^ × 3 mm^3^ × 3 mm^3^, and finally, a Gaussian kernel was used for spatial smoothing (σ = 2 mm).

## Results

**FIGURE 5:**
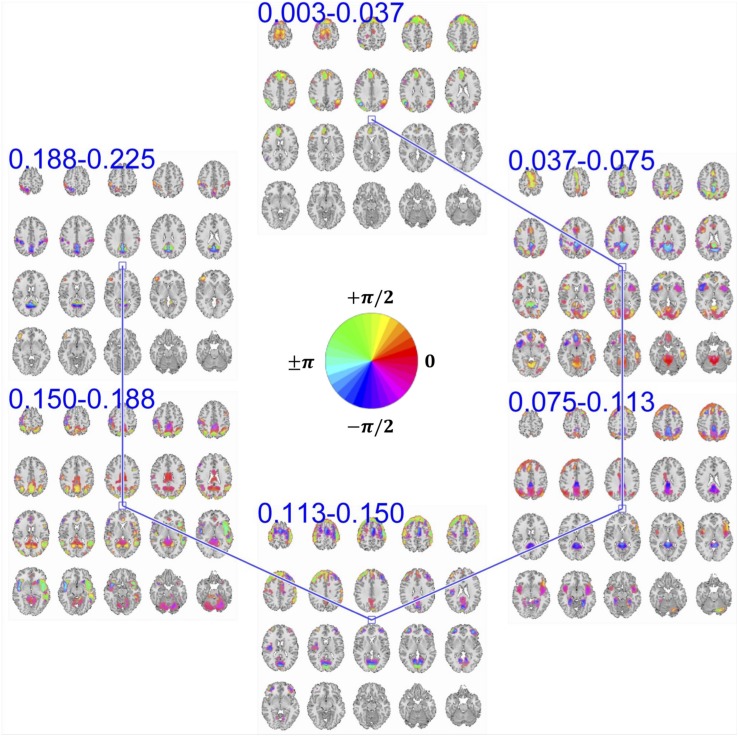
An example of summarization bases in voxel space comprising six frequency-specific spatial maps. Each spatial map depicts maps of voxels with a significant level of activation, which is determined by thresholding voxels activity amplitudes using cluster-level threshold. In addition to the amplitude, each voxel has an estimation of phase of the activity which when is compared to the phase of another voxel, it determines the relative synchrony of co-activation of the two voxels. For example, when two voxels have the same phase (i.e., their phase is encoded with the same color derived from the provided color-coding), it implies zero-lagged co-activation (a.k.a., positive correlation). Similarly, when the phase difference is at its maximum, it implies a negative correlation. In the middle of the figure, we represent the color map we use to encode the phase information.

Our approach provides a unique opportunity to study brain functional segregation and integration across different frequency ranges, which enables a more precise model of brain function concerning different HRF modulations. The key contribution of this work is the estimation of frequency-specific spatial maps corresponding to the summarized bases of input data in the frequency-augmented voxel space. Here, each basis constitutes six band-specific spatial maps. Figure 5 demonstrates an example of such a basis obtained from the proposed approach. First, decomposition was set to solve for 75 of such a basis. Because we are working in the time-frequency domain, the activation level of each voxel in a given spatial map is a complex value. The magnitude of the complex-valued activation represents the intensity of activation. At the same time, its phase encodes degree of relative delay or advance of the activation with respect to a reference activity which is defined by the choice of zero in the time domain. Because the choice of reference along time domain is arbitrary, phase information becomes meaningful when compared between multiple activities.

Two voxels that have the same phase value implies a positive correlation between their temporal activity patterns. If these two voxels have different phases of activation, the temporal activity of one voxel is delayed/advanced with respect to the other voxel. When the phase difference between the two voxels is at its maximum (180°), an anti-correlative relationship exists between them. In the provided spatial maps that represent the phase, we use a circular color-coding presented in the center of Figure 5.

MCCA enables the estimation of frequency-specific spatial maps of each basis in a way that maximizes the dependence between their corresponding TCs. However, a maximum dependence criterion does not guarantee that the temporal dependence between spatial maps is significant. To evaluate the significance of temporal dependence between these spatial maps, we represent the null distribution of the dependence as zero dependence by randomly shuffling each time-course’s time-point and estimating pair-wise correlation of these band-specific TCs over 100 repetitions. Note that TCs are complex-valued and the correlation is estimated as the conjugate inner product of two TCs and the null-distribution is created by taking the absolute value of the inner product. We mark dependence between spatial maps as significant if their pair-wise temporal correlation value is greater than 99% of the null-distribution. Pair-wise dependence, which surpasses this significance test, is indicated with a solid line between the corresponding spatial maps. As seen in Figure 5, spatial maps with neighboring bands have a significant temporal dependency with each other, a general observation across all estimated bases.

However, ambiguity exists regarding the nature of the temporal dependence between neighboring frequency bands, due to overlap in the frequency spectrum. The overlapping spectrum causes the neighboring spatial maps to contain a degree of shared temporal information across separated parts of the spectrum, thereby resembling cross-frequency dependence. Nevertheless, adjacent frequency bands enable us to observe the evolution of spatial maps in frequency at a higher resolution.

**FIGURE 6:**
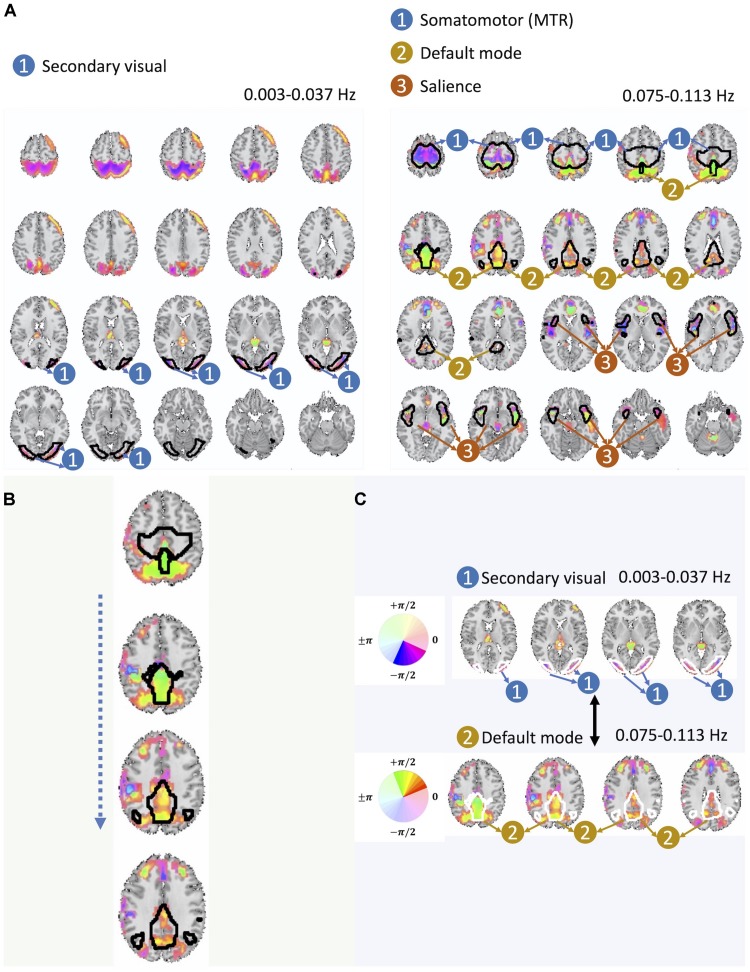
**(A)** Here we present one out of the two pairs of spatial maps with a significant cross-frequency dependence belonging to non-adjacent frequency bands (the one on the left belongs to the frequency range of 0.003–0.037 Hz and the one on the right belongs to the frequency range of 0.075–0.113 Hz) as an evidence of cross-frequency dependence between different areas of the brain captured by fMRI. **(B)** Spatial variation of the phase within the DMN. **(C)** Varying degree of lagged and the cross-frequency correlation between DMN (bottom) and visual networks (top) captured by the phase information.

Our analysis further identifies two instances of significant cross-frequency dependence between pairs of spatial maps belonging to the same basis that occur between non-overlapping frequency bands, which we have shown in Figure 6A. For each spatial map, we only show a map of voxels whose estimated amplitude survive significance threshold as well as cluster-level threshold (details of thresholding step is provided at the end of this section). We also provide color-coded phase information of these voxels along with the labeling of groups of voxels by their identified corresponding large-scale brain networks. Twelve large-scale brain networks were identified using low-order ICA on the dataset, following the same analysis steps and choice of parameters as our earlier works (Iraji et al., 2016; Iraji et al., 2019a). To identify the voxel associated with brain networks, the threshold was set at Z > 1.96. The identified brain networks include the default mode network (DMN), salience network, (dorsal) attention, subcortical, auditory, cerebellar, left and right frontoparietal, somatomotor (MTR), primary visual, and secondary visual. If the overlap between a given spatial map and each of these twelve networks was greater than 60%, we assign the corresponding label to that spatial map.

Before making a further interpretation of the estimated bases and corresponding spatial maps, we investigated the relative delayed functional connectivity at a better resolution by using phase information, in contrast to modeling positive and negative correlation. For instance, in Figure 6A and more visible in Figure 6B, the area that has been labeled as the DMN has an evident variation of phase within itself (varying along *z*-axis), which provides evidence of lagged activation of different areas of the DMN. Without the phase information, such intra-network lagged activations could not be observed. Moreover, there is interesting evidence of cross-frequency dependence between the visual network (occurring at relatively lower frequency band i.e., 0.003–0.037 Hz) and the DMN (occurring at relatively higher frequency band, 0.075–0.113 Hz) (Figure 6A and more visible in Figure 6C).

**FIGURE 7:**
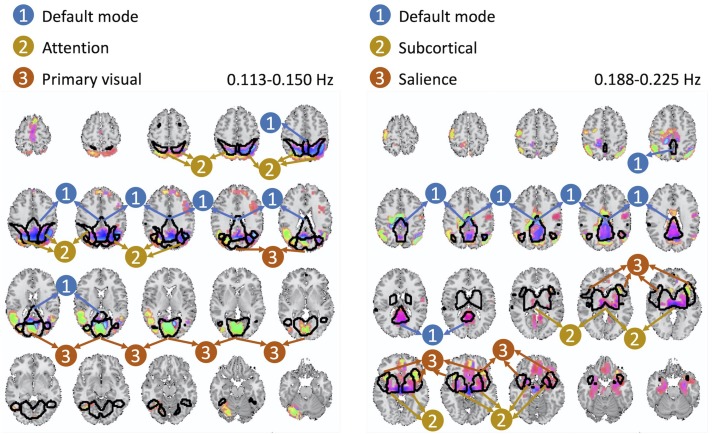
The second pair of spatial maps exhibiting significant dependence with regard to their corresponding temporal activity occurring at non-overlapping frequency bands.

The cross-frequency dependence between the visual network provides evidence of variation in the phase of cross-frequency dependence. Although existing studies report negative correlation between the visual network and the DMN (Uddin et al., 2009; Allen et al., 2011; Iraji et al., 2019a), our study provides new information on the true nature of such dependence in respect to both the frequency at which such dependence occurs as well as the degree of the actual lag in the dependence which varies over different parts of both the visual network and the DMN. Similar observations can be made from the second pair of spatial maps with significant cross-frequency dependence shown in Figure 7. However, future research should attend to understand neurobiological grounds of the observed lagged and cross-frequency connectivity, as this was outside of the scope of the current study.

We observe many instances of cross-frequency dependence between neighboring frequency bands and cross-frequency dependence between non-neighboring and more distant bands. Thus, we sought to determine whether cross-frequency dependence varied across subjects. To assess variation in age and gender, we perform multivariate comparisons as described in Allen et al. (2011) while controlling for motion parameters.

More precisely, we measure the cross-frequency dependence between each pair of spatial maps that met the threshold of significance for each subject. Each individual’s data provides a sample for a multivariate response variable, in which the dimensions of the variables are the selected measured cross-frequency dependence. Since this multivariate response variable is low-rank, we perform a PCA-based dimension reduction on this data (Allen et al., 2011) and reduce the dimension to 70 (retaining 80% of all variations in the data). We then employ the MANCOVAN toolbox implementation^4^ of stepwise backward explanatory variable selection which includes age, gender, and motion parameters. The reduced set of variables includes gender and motion parameters.

**FIGURE 8:**
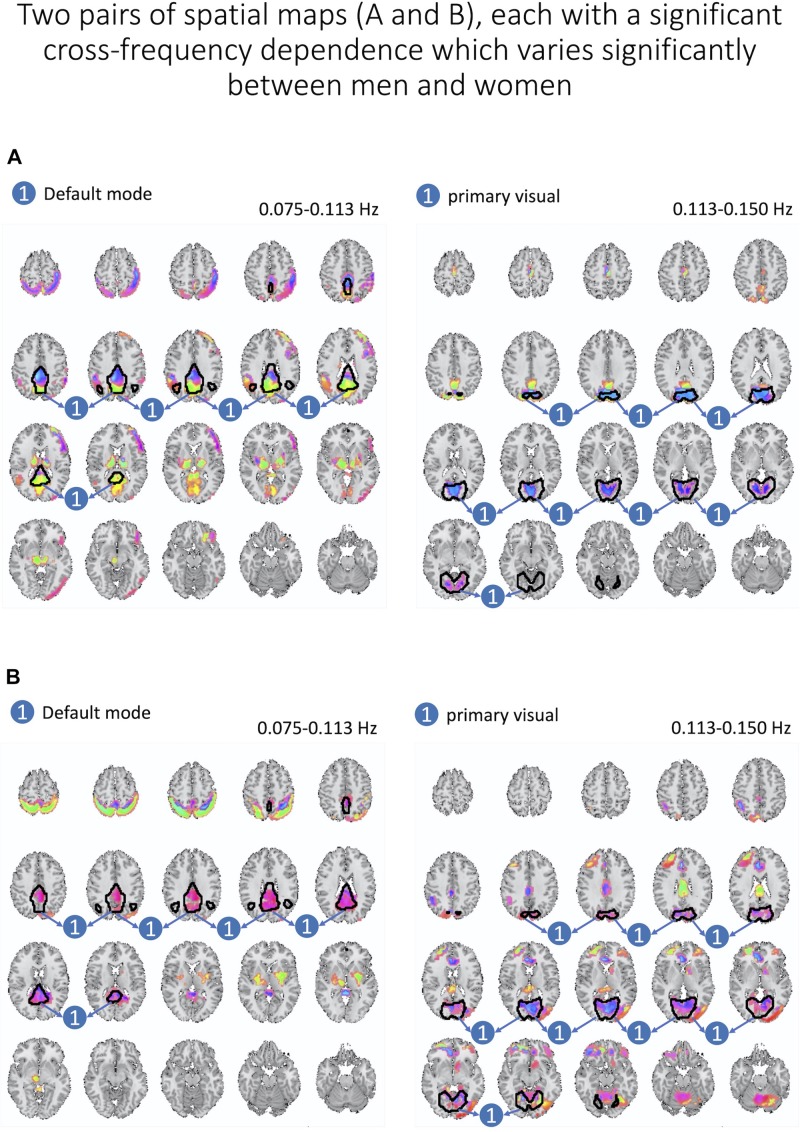
Here, we present two pairs of spatial maps **(A, B)**, in which each pair represents two spatial maps (left and right) belonging to two different frequency intervals but with a significant dependence between each other. Furthermore, cross-frequency dependence varies significantly between women and men. In both pairs, women have a stronger degree of dependence across labeled areas of each spatial map.

Next, we assess whether gender moderated each cross-frequency dependence after regressing out motion parameters and after correcting for multiple comparisons based on FDR adjustments of estimated *p*-values. We identified two pairs of spatial maps depicted in Figure 8 with cross-frequency dependence being strongly correlated to the gender with FDR-adjusted *p*-values of 0.007 and 0.008. In both cases, women showed stronger cross-frequency dependence than men.

Finally, each spatial map shows only the voxels with amplitudes that survive the significance threshold as well as the cluster-level threshold of size 40. To estimate the significance threshold, we use back-reconstruction to derive subject-level spatial maps from group-level bases (Erhardt et al., 2011). Initially, our proposed summarization approach estimates spatial maps as bases in voxel space that are shared among all subjects. Using back-reconstruction allows us to assess subject-specific spatial maps and, consequently, the distribution of each voxel’s activation level within each spatial map. From this distribution, we estimate the *F*-score to determine the region of significance. We use the *F*-score for each voxel because the values are in the complex domain and are represented by both real and imaginary parts as a 2-d vector. For each spatial map, we multiply 0.25 with the maximum *F*-score to threshold the *F*-score and remove clusters less than 40 voxels.

## Discussion

In this work, we propose a novel summarization approach based on dependence as a general function of frequency. Previous work in fMRI has captured dependence between the activity of different areas of the brain as a function of frequency but was limited to in-between frequency dependence (Cordes et al., 2001; Chang and Glover, 2010).

Methods for summarization of brain data in the augmented time and frequency domain have been lacking across fMRI studies, as well as other imaging modalities, such as EEG and MEG. EEG and MEG studies have utilized cross-frequency dependence, referred to as cross-frequency coupling (Cohen, 2008; Canolty and Knight, 2010), but these analyses have not yet been extended to summarization approaches. A strength of the proposed approach lies in its applicability. Specifically, our approach does not restrict any assumptions on the nature of the data and thus also has a strong potential of being leveraged for brain research in modalities beyond fMRI.

The technical novelty of the proposed approach is the way we concatenate the frequency-specific data for the first step of summarization. Explicitly, we model dependence as a function of one frequency, which covers both in-between frequency dependence as well as dependence that occurs over a range of frequencies (aggregate in-between dependence). Additionally, we adjust the summarization step to also account for cross-frequency dependence by using MCCA.

Employing MCCA during summarization represents a novel technical approach. As explained earlier, this step finds a new set of bases (${\text{B}}_{\text{i}}^{\text{T}}$), one for each frequency band for the subspace originally derived from the preceding ICA. Consequently, MCCA transforms bases in voxel space estimated by ICA. The main property of this transformation lies in the following two equations:

1. $\forall iE\left(\text{S}{\left(\text{t}\right)}_{\text{i}}^{{}^{\prime }},S{\left(\text{t}\right)}_{\text{i}}^{{}^{\prime }}\text{T}\right)=\text{I}$ where I is identity matrix.
2. $\forall \text{i}\neq jE\left(\text{S}{\left(\text{t}\right)}_{\text{i}}^{{}^{\prime }},S{\left(\text{t}\right)}_{\text{j}}^{{}^{\prime }}\text{T}\right)=\Lambda$ where Λ a diagonal matrix (please refer to Eq. 6 for detailed formulation).

The first equation indicates that MCCA transforms the initial spatial parcellation of the brain, which is derived from the spatial ICA and is shared between frequency bands, into new spatial maps which are band-specific and whose time-courses are uncorrelated with other spatial maps’ time-courses at the corresponding band. This property resembles work referred to as meta-state analysis (Cohen, 2008; Miller et al., 2015; Yaesoubi et al., 2015b), which assumes that connectivity of the brain constitutes of multiple stationary (temporally-static) connectivity patterns. Simultaneously, (temporally) dynamic connectivity (also referred to as dynamic coupling) exists across these stationary connectivity patterns. In the context of static analysis, dependence is minimized, consistent with constraining the correlation matrix with a diagonal constant. The second constraint is simply an extension of the first constraint, accounting for the cross-frequency connectivity between corresponding transformed spatial maps across frequency bands.

### Limitations and Future Work

The major limitation of our work stems from the fact that dependence and summarization, in general, is computed in linear space. Both PCA and ICA in the first step of the proposed framework as well as the MCCA from the second step measure linear dependence (i.e., correlation). Additionally, our choice of parameters could influence the results. We relied on prior studies (Allen et al., 2014; Yaesoubi et al., 2015a) to choose the dimension of the subspace derived from the PCA-based dimension reduction step. Future research should investigate whether different number of dimensions could impact results. Similarly, we chose six frequency bands to resemble the bands used in Yaesoubi et al. (2015a) in order to estimate frequency-specific and dynamic connectivity states of fMRI. However, in prior work time-frequency transformation was applied to the ICA spatial map TCs, which is integrated over a weighted collection of voxel time-series. This is in contrast with the current study, in which we applied the transformation directly to voxel time-series to provide more detailed resolution of the frequency bands.

Currently, we capture spatial maps based on stationary dependence. One important direction for future research is to capture dynamic dependence similar to sliding-window approaches or as mentioned meta-states analyses (Calhoun et al., 2014).

Our work is based on minimal assumptions regarding the nature of the input data and is easily applicable to other imaging modalities such as EEG or MEG. Also, our observation that cross-frequency dependence varies based on participant characteristics (e.g., gender) demonstrates the potential of the approach. This can be extended in future application to clinical research studies to investigate the potential of these frequency-dependent effects across other types of participant characteristics, such as differences in mental and physical health.

## Data Availability Statement

The datasets for this manuscript are not publicly available because Raw imaging data is not available yet as it’s pooled data from about 30 different investigators at MRN and some of the studies do not have subjects sign data sharing agreements. Requests to access the datasets should be directed to vcalhoun@mrn.org.

## Ethics Statement

Subjects were given written informed consent, following institutional guidelines approved by 245 Institutional Review Board of the University of New Mexico.

## Author Contributions

All authors listed have made a substantial, direct and intellectual contribution to the work, and approved it for publication.

## Conflict of Interest

The authors declare that the research was conducted in the absence of any commercial or financial relationships that could be construed as a potential conflict of interest.

## Footnotes

1 Throughout this work, by ICA we are referring to spatial ICA.

2 For real data we are using 6 separate frequency bands, as indicated previously.

3 http://www.fil.ion.ucl.ac.uk/spm/

4 http://www.mathworks.com/matlabcentral/fileexchange/27014-mancovan
